# Towards optimization of plant cell detection in suspensions using impedance-based analyses and the unified equivalent circuit model

**DOI:** 10.1038/s41598-021-98901-0

**Published:** 2021-09-29

**Authors:** Kian Kadan-Jamal, Aakash Jog, Marios Sophocleous, Julius Georgiou, Adi Avni, Yosi Shacham-Diamand

**Affiliations:** 1 Department of Materials Science and Engineering, Faculty of Engineering, Tel Aviv University, 69978 Tel Aviv, Israel; 2 Department of Physical Electronics, School of Electrical Engineering, Faculty of Engineering, Tel Aviv University, 69978 Tel Aviv, Israel; 3 Department of Electrical and Computer Engineering, EMPHASIS Research Center, University of Cyprus, 1678 Nicosia, Cyprus; 4 School of Plant Sciences and Food Security, Tel-Aviv University, Tel Aviv, Israel

**Keywords:** Sensors and probes, Biosensors, Plant sciences

## Abstract

An improved approach for comparative study of plant cells for long term and continuous monitoring using electrical impedance spectroscopy is demonstrated for tomato and tobacco plant cells (MSK8 and BY2) in suspensions. This approach is based on the locations and magnitudes of defining features in the impedance spectra of the recently reported unified equivalent circuit model. The ultra-wide range (4 Hz to 20 GHz) impedance spectra of the cell lines were measured using custom probes, and were analyzed using the unified equivalent circuit model, highlighting significant negative phase peaks in the ~ 1 kHz to ~ 10 MHz range. These peaks differ between the tomato and tobacco cells, and since they can be easily defined, they can potentially be used as the signal for differentiating between different cell cultures or monitoring them over time. These findings were further analysed, showing that ratios relating the resistances of the media and the resistance of the cells define the sensitivity of the method, thus affecting its selectivity. It was further shown that cell agglomeration is also an important factor in the impedance modeling in addition to the overall cell concentration. These results can be used for optimizing and calibrating electrical impedance spectroscopy-based sensors for long term monitoring of cell lines in suspension for a given specific cell and media types.

## Introduction

The global population is expected to exceed 9 billion by 2050, demanding an increase in food production by approximately 70%^1,2^. However, while the population increases, the arable land size is gradually decreasing, further aggravating the need for more efficient agricultural methods^3–5^. Monitoring methods for use in precision agriculture are necessary to balance the increasing demands and the reducing land availability^6–8^. In order to achieve sustainability, it is important to gather data and monitor farming parameters such as soil quality, plants, crops and other environmental parameters^9–16^. Data driven agriculture requires research and development of low-cost, field-deployable sensors that can be easily interfaced to the internet and Internet of Things (IoT) compatible^17^. Therefore, electrical sensors with low-cost electronics, which can be mass produced, are being investigated.

One family of such sensors is based on Electrical Impedance Spectroscopy (EIS), and uses commonly available impedance measurement systems. EIS has been widely used in biomedical applications^18,19^, with a variety of cells and tissues. Thus, there exists a very thorough background for this technique, both in theory and in practice. In particular, EIS can be used in the field of agriculture and food, for assessment of plant cell and tissue conditions^20–24^. The EIS spectra of the plant cell or tissue can be represented by an equivalent electrical circuit, using lumped components. The electrical topology of such a model can be decided arbitrarily based on optimal fitting, or be based on the physical properties and the macro and micro structures of the cells, tissues, and organs^25,26^. The impedance spectra of plant cells are affected by physical parameters of the cells, such as the shape of the cells, electrical properties of the cell wall, plasma membranes, cytoplasm, and intra and extracellular conductivities^27,28^. Additionally, EIS can be used to demonstrate a close relationship between the dielectric properties of suspended cells, like capacitance, and its biomass concentration^29–31^. EIS can also be used to estimate the thickness of the cell wall and plasma membrane by properly modelling the various volume fractions of the cell suspensions^21,27,28,32^. A unified equivalent circuit model for plant cells has been recently reported^33,34^ for an ultra-wide frequency range (4 Hz–20 GHz). The unified model was used to fit experimentally obtained EIS spectra of tomato cells (MSK8) in Murashige and Skoog (MS) media. However, further investigation into the dependence of this method on the properties of the cells and media is required. Although the reported equivalent circuit model has been shown to provide accurate fits to the measurements, the way to use that model is not investigated. In order for that model to become useful in real-time monitoring of plant health, the relationships between the model’s components with plant physiology should be identified in a systematic manner.

Hence, a novel study on two different cell types in two different media has been presented in this work. This work attempts to build the foundations for relating the parameter variations of the model with plant physiological changes but also looking into how those relationships can change from one type of plant to another. Furthermore, this work is an attempt to show that optimization of monitoring parameters is feasible by varying specific media parameters. Suspensions of tomato cells (MSK8) and tobacco cells (BY2) in Murashige and Skoog (MS) and phosphate buffer (PB) media were studied. The experimentally observed spectra were fitted to the unified model. Although all data exhibited a good fitting with the model, a few fundamental differences were observed in the measurements in the range of ~ 1 kHz to ~ 10 MHz. The most pronounced differences were those in the height and locations of peaks in the phase spectra. Therefore, a new analytical approach for characterization of the concentration and type of cells in a cell suspension, has been proposed. This approach is based on the locations and heights of peaks in the phase of the impedance spectra, and the heights of plateaus in the gain of the impedance spectra. The theoretical analyses are presented and discussed critically, and are applied to the experimentally obtained data in order to demonstrate their efficacy with regards to discrimination between cell and medium types.

## Materials and methods

### Preparation and analysis of cells

#### Cell cultures

Tomato (*S*. *lycopersicum* cv Mill.; line MSK8^35^) and tobacco (*N*. *tabacum* cv BY2^36^) cell suspension cultures were grown in Murashige and Skoog (MS)^37^ media including vitamins (Duchefa Biochemie) in 250 mL flask with 100 mL liquid, supplemented with 30 g/L sucrose, 1 mg/L 2,4-dichlorophenoxyacetic acid (2,4-D) and 0.1 mg/L kinetin, which was set to pH 5.7. Both cell lines were prepared and grown in-house at the School of Plant Sciences and Food Security, Tel Aviv University, following standard procedures^35,36^. The cell cultures were centrifuged at 25 °C in the dark, at approximately 100 rpm. Sub-culturing was performed every 2 weeks. MSK8 cells were used 14–20 days after sub-culturing and BY2 cells were used 4–6 days after weekly sub-culturing. The cells samples were diluted before the experiment in fresh MS or PB media [0.1 M] at pH 5.8^33^.

### EIS measurements

EIS measurements were carried out in the frequency range of 4 Hz to 20 GHz using multiple instruments. More details about the setup and tools used, and their error margins can be found in^34^. For each experiment, cells were filtered out from the growth media, and then re-suspended in fresh media before the beginning of each measurement. The suspensions were diluted down to the required concentrations using fresh media. All measurements were performed at room temperature (25 °C). Each experiment was repeated 3–5 times, and the differences between the obtained impedance spectra were observed to be well within the error margins of the equipment used^33^.

## Results and discussion

### Electrical modelling

**Figure 1:**
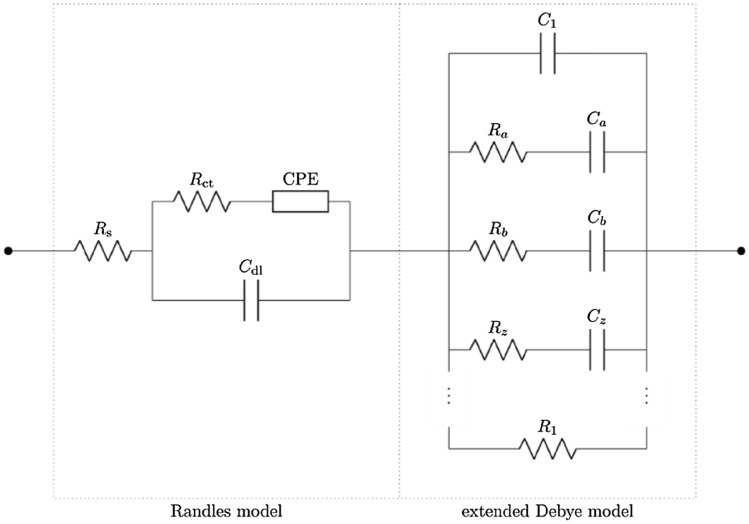
The unified equivalent circuit model of the cell-line suspension, and in the extended Debye model section^33^.

Based on previously published works, the cell suspensions can be modelled using the unified Randles–Debye model (see Fig. 1)^33^. In this model, $R_{s}$, $R_{ct}$, $C_{dl}$, and the CPE as are defined in a standard Randles model, *R* represents the resistance of the cells, $R_{1}$ represents the resistance of the solution, $C$ represents the capacitance due to the presence of cells in the suspension, and $C_{1}$ represents the capacitive effects of water polarization.

An impedance spectrum obtained from the simplified version of the unified Randles–Debye model, consists of three distinct dispersions corresponding to the three capacitors $C_{dl}$, C, and $C_{1}$. Of these, the first represents the double layer capacitance and the remaining two represent the imaginary parts of the Debye dispersion due to dielectric relaxation at high frequencies. Each dispersion can be represented by a simple rational first order polynomial function with one pole and one zero, such that the pole frequency is less than that of the zero. Hence, these dispersions correspond to 3 poles and 3 zeroes, thus creating 7 regions in the impedance spectrum. For the sake of simplicity, the Warburg element in the Randles model has been ignored in this analysis since it only comes into effect at very low frequencies (i.e. below the lowest studied frequency of 4 Hz). The analysis uses the notation $\omega_{ij}$ to denote the corner angular frequency (either corresponding to a pole or to a zero) between regions $i$ and $j$. The angular frequency is related to the frequency as $\omega_{ij} = 2\pi f_{ij}$.

**Figure 2:**
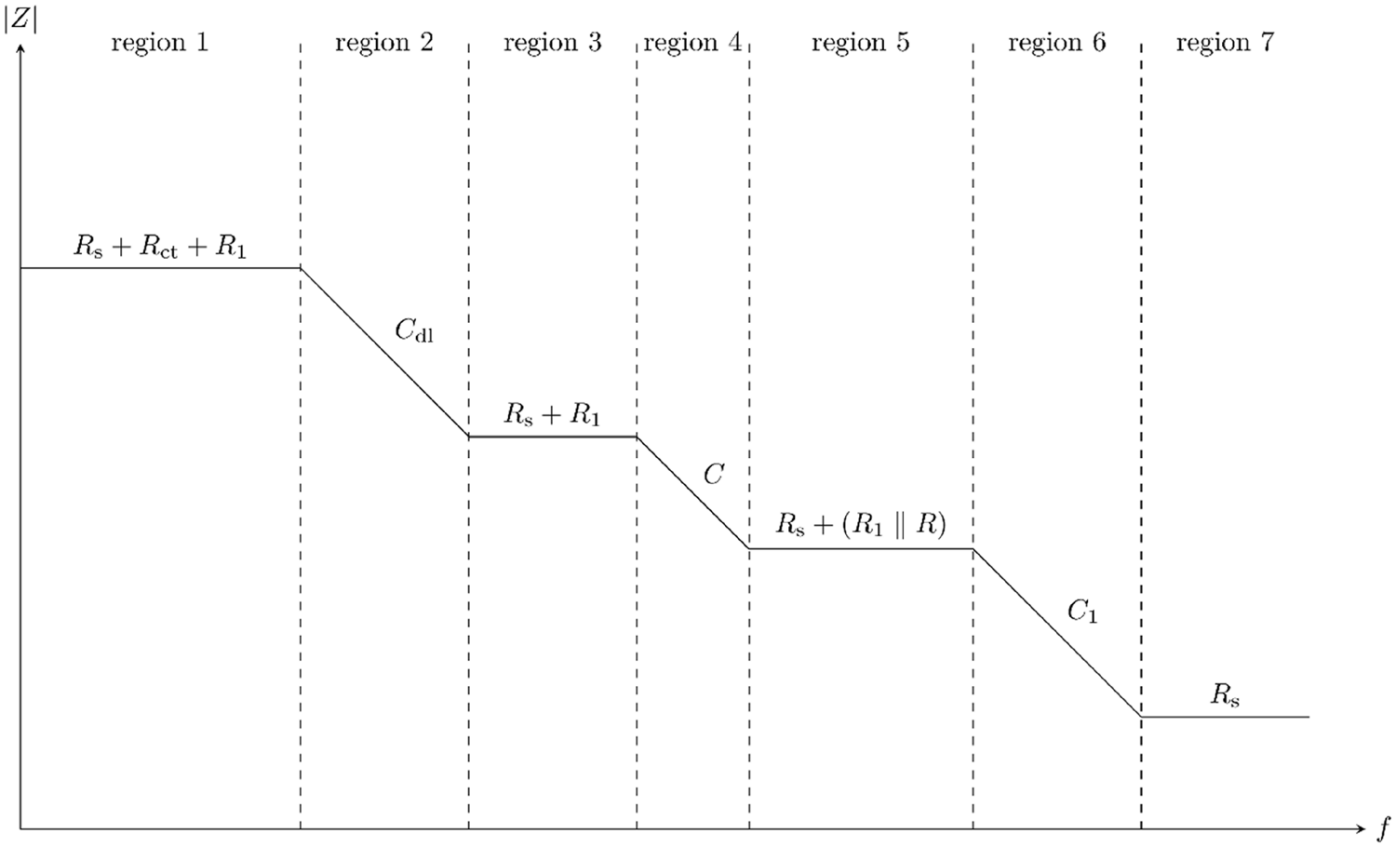
A schematic drawing based on the asymptote-based approximation of the impedance spectra of the simplified unified equivalent circuit model for cell suspensions.

Figure 2 shows the asymptote-based impedance magnitude spectrum of the unified equivalent circuit model on logarithmic axes^33^. Region 1 corresponds to the lowest range of frequencies, in which all three capacitors have very high impedance (relative to their components in parallel with them). Hence, the magnitude of the impedance is approximately $R_{s} + R_{ct} + R_{1}$. The first pole, at the angular frequency $\omega_{12}$, is determined by the largest capacitor, i.e. $C_{dl}$. Hence, the pole occurs at the frequency at which the impedance of $C_{dl}$ matches $R_{s} + R_{ct} + R_{1}$.

In region 2, the charge transfer resistance ($R_{ct}$) in the Randles model is shunted by the double layer capacitance ($C_{dl}$). In this region, the magnitude of the impedance falls at a rate of − 20 dB/dec. The zero due to $C_{dl}$ corresponds to the frequency at which the impedance, corresponding to the double layer capacitance, becomes negligibly small compared to $R_{s} + R_{1}$.

The second-largest capacitor, i.e. C, behaves in the opposite manner. Its impedance comes into effect once it becomes comparable to $R_{s} + R_{1}$, i.e. beyond $\omega_{34}$. Once the magnitude of its impedance drops to a sufficiently small value, the impedance of the R–C branch in the Debye model is governed by R. Hence, in region 5, the magnitude of the impedance is approximately $R_{s} + R_{1} \parallel R$.

The smallest capacitor, i.e. $C_{1}$, behaves in a manner similar to $C_{dl}$, by shunting the other branches of the Debye model. The frequencies and impedance magnitudes corresponding to the poles and zeros are as in Table S1. The frequency dependence of each dispersion can be described using a bilinear complex function $\frac{{1 + j\frac{\omega }{{\omega_{{{\text{zero}}}} }}}}{{1 + j\frac{\omega }{{\omega_{{{\text{pole}}}} }}}}$ where $\omega_{{{\text{pole}}}}$, the angular frequency corresponding to the pole, is smaller than $\omega_{{{\text{zero}}}}$, the angular frequency corresponding to the zero. On a logarithmic frequency axis, the phase of this bilinear complex function has a bell-shape, with the negative phase peak occurring at a frequency which is the geometric mean of the pole and zero frequencies, i.e. $\omega_{{{\text{peak}}}} = \sqrt {\omega_{{{\text{pole}}}} \cdot \omega_{{{\text{zero}}}} }$. Thence, the minimum phase (i.e. the phase at $\omega_{{{\text{peak}}}}$), is determined by:

1 $$ \omega_{{{\text{min}}\;{\text{phase}}}} = \arctan \left( {\sqrt {\frac{{\omega_{{{\text{pole}}}} }}{{\omega_{{{\text{zero}}}} }}} } \right) - \arctan \left( {\sqrt {\frac{{\omega_{{{\text{zero}}}} }}{{\omega_{{{\text{pole}}}} }}} } \right) $$

Therefore, the negative phase peaks in regions 4 and 6 correspond to the frequency pairs $(\omega_{34} ,\;\omega_{45} )$ and $(\omega_{56} ,\;\omega_{67} )$, respectively. Henceforth, the negative phase peaks in regions 4 and 6 are referred to as $\varphi_{4}$ and $\varphi_{6}$, respectively, and the frequencies corresponding to these peaks are referred to as $f_{4}$ and $f_{6}$, respectively.

**Figure 3:**
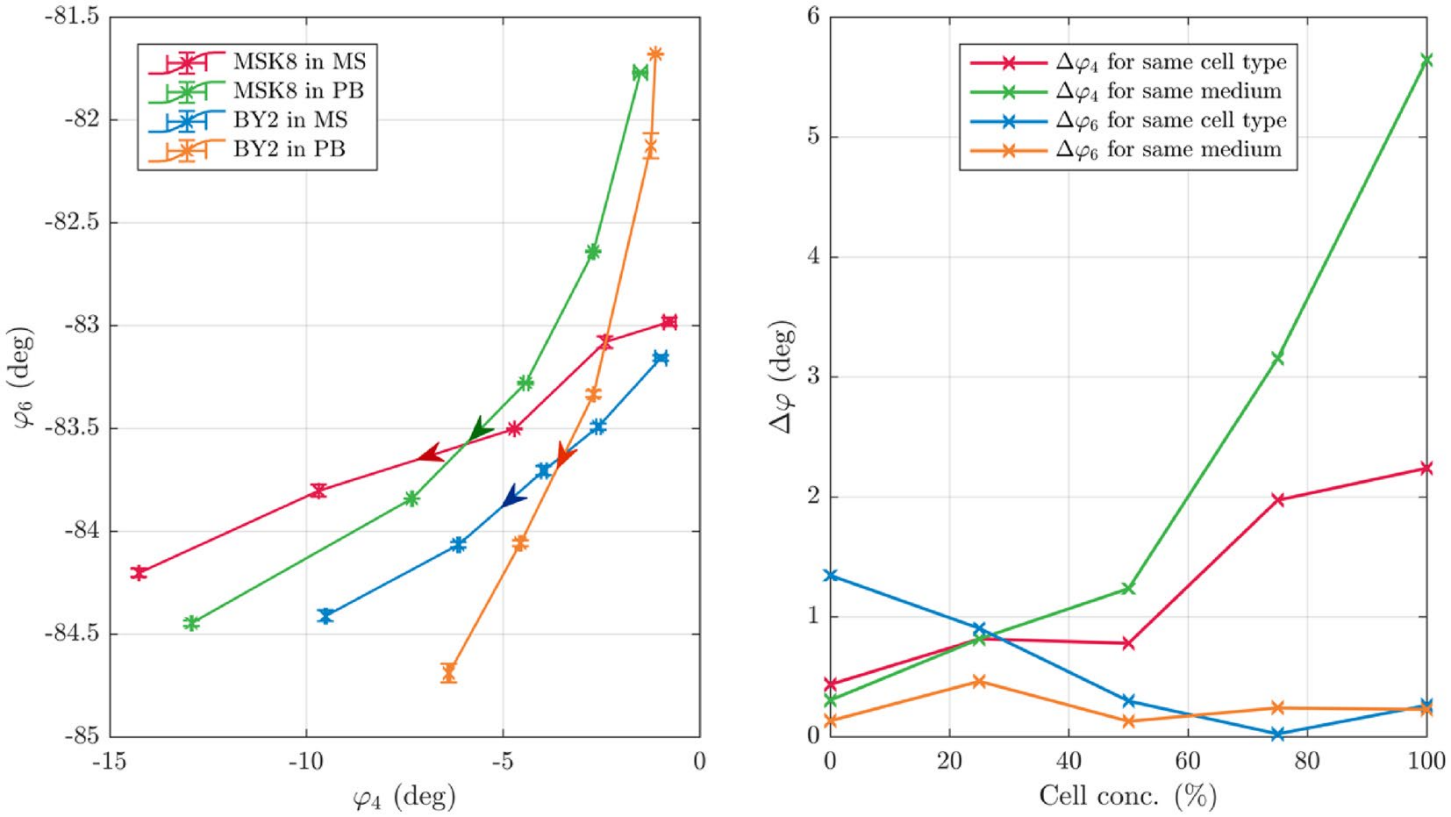
(left) $\varphi_{4}$ vs $\varphi_{6}$ for all four suspensions across the entire range of cell concentration, with the arrows indicating increasing cell concentration, and error bars indicating min–max bounds (right) mean deviation in $\varphi_{4}$ ($\Delta \varphi_{4}$) for all pairs of suspensions with the same cell-type, and for all pairs of suspensions with the same media.

The experimentally obtained impedance spectra for all combinations of cells and media were fitted to the unified equivalent circuit model (see Fig. 3)^33^. The locations and heights of the two aforementioned phase peaks were calculated based on the fitted parameters and the above analytically obtained expressions. In order to study the impact of cell and medium type on $\varphi_{4}$ and $\varphi_{6}$, the two were plotted against each other, for all four combinations of cell types and media.

Figure 3 (left) shows $\varphi_{4}$ and $\varphi_{6}$, derived from the experimental spectra, for all four suspensions across the entire range of cell concentration. As expected based on previous work^33^, suspensions with higher concentrations have larger negative phase peaks, i.e. higher $\varphi_{4}$ and $\varphi_{6}$. Moreover, for low cell concentrations, the minimum phases for a given suspension are closer to those for suspensions with the same media type (e.g. MSK8 in MS and BY2 in MS). Conversely, for high cell concentrations, the minimum phases are closer for suspensions with the same cell type (e.g. MSK8 in MS and MSK8 in PB).

Figure 3 (right) shows the mean deviation in $\varphi_{4}$ ($\Delta \varphi_{4}$) for all pairs of suspensions with the same cell-type, and for all pairs of suspensions with the same media. As the concentration of cells in the suspensions increases, $\Delta \varphi_{4}$ increases for both of types of neighbors. However, the values and rate of increase in $\Delta \varphi_{4}$ for suspensions with the same media are much larger than that for suspensions with the same cell-type. This is consistent with the expectation that $\varphi_{4}$ is determined primarily by the cells (and not the media), and thence the deviation in $\varphi_{4}$ should be lower for suspensions consisting of the same cell type^33^.

In order to further analyze these behaviors, an analytical approach was adopted.

### Mathematical modelling and detection optimization

The impedance spectra—and hence the extracted parameters corresponding to the phase peaks and magnitude plateaus—depend not only on the absolute values of the resistances in the unified model, but also on the ratio between them.

Therefore, in order to analyze the dependence on these ratios, let $R = kR_{1}$ and let $R_{s} = pR_{1}$. Hence, $\omega_{4}$ and $\omega_{6}$ vary with *k* and *p* according to:

2 $$ \omega_{4} = \frac{1}{{CR_{1} }}\sqrt {\frac{1}{k(k + 1)}} $$

3 $$ \omega_{6} = \frac{1}{{C_{1} R_{1} }}\sqrt {\frac{1}{{p\left( {p + \frac{k}{k + 1}} \right)}}} $$

Similarly, $\varphi_{4}$ and $\varphi_{6}$ vary with *k* and *p* according to:

4 $$ \varphi_{4} = \arctan \left( {\sqrt {\frac{k}{k + 1}} } \right) - \arctan \left( {\sqrt {\frac{k + 1}{k}} } \right) $$

5 $$ \varphi_{6} = \arctan \left( {\sqrt {\frac{p}{{p + \frac{k}{k + 1}}}} } \right) - \arctan \left( {\sqrt {\frac{{p + \frac{k}{k + 1}}}{p}} } \right) $$

Both $\varphi_{4}$ and $\varphi_{6}$ are independent of the capacitances $C$ and $C_{1}$, and depend only on the ratios of the resistances. In particular, for an infinitesimally small $k$, i.e. if $R$ is significantly smaller than $R_{1}$, then $\varphi_{4}$ approaches − 90°. However, practically for most cell types and media, $R$ is larger than $R_{1}$. Hence, $\varphi_{4}$ is bounded by approximately − 20° from below.

Similarly, as $R_{s}$ is orders of magnitude smaller than $R_{1}$, $\varphi_{6}$ is close to − 90° (and in particular is bounded from above by approximately − 74° for $k = 1$ and $p = 0.01$).

Based on the above equations, $\varphi_{4}$ depends only on $k$, whilst the capacitance due to the cells only affects $f_{4}$. It would have been expected that $f_{4}$ would be proportional to the concentration of the cells. However, Figure S7 (left) shows that the relationship is somewhat random for $f_{4}$ whereas Figure S7 (right) shows that there exists a much clearer relationship between $f_{6}$ and the cell concentration. The microscopic images of the cells shown in Figures S6 show that cells agglomerate significantly, an effect that has a more significant influence on the capacitance compared to the effect of concentration. On the other hand, $f_{6}$ shows as much clearer relationship on the cell concentration since it depends mostly on the effect of the media (MS or PB).

Therefore, in order to amplify the effects of cell concentration on $\varphi_{4}$, $k$ should be as small as possible, i.e. $R$ should be as close to or smaller than $R_{1}$. Solving with $k$ and $p$ as above, the magnitude of the impedance in region 5 is:

6 $$ \left| Z \right|_{5} = R_{1} \left( {p + \frac{k}{k + 1}} \right) $$

For a given cell suspension, the deviation in $\left| Z \right|_{5}$ with respect to the medium is defined by:

7 $$ d = \frac{{\left| Z \right|_{{5{\text{suspension}}}} }}{{\left| Z \right|_{{5{\text{medium}}}} }} $$

**Figure 4:**
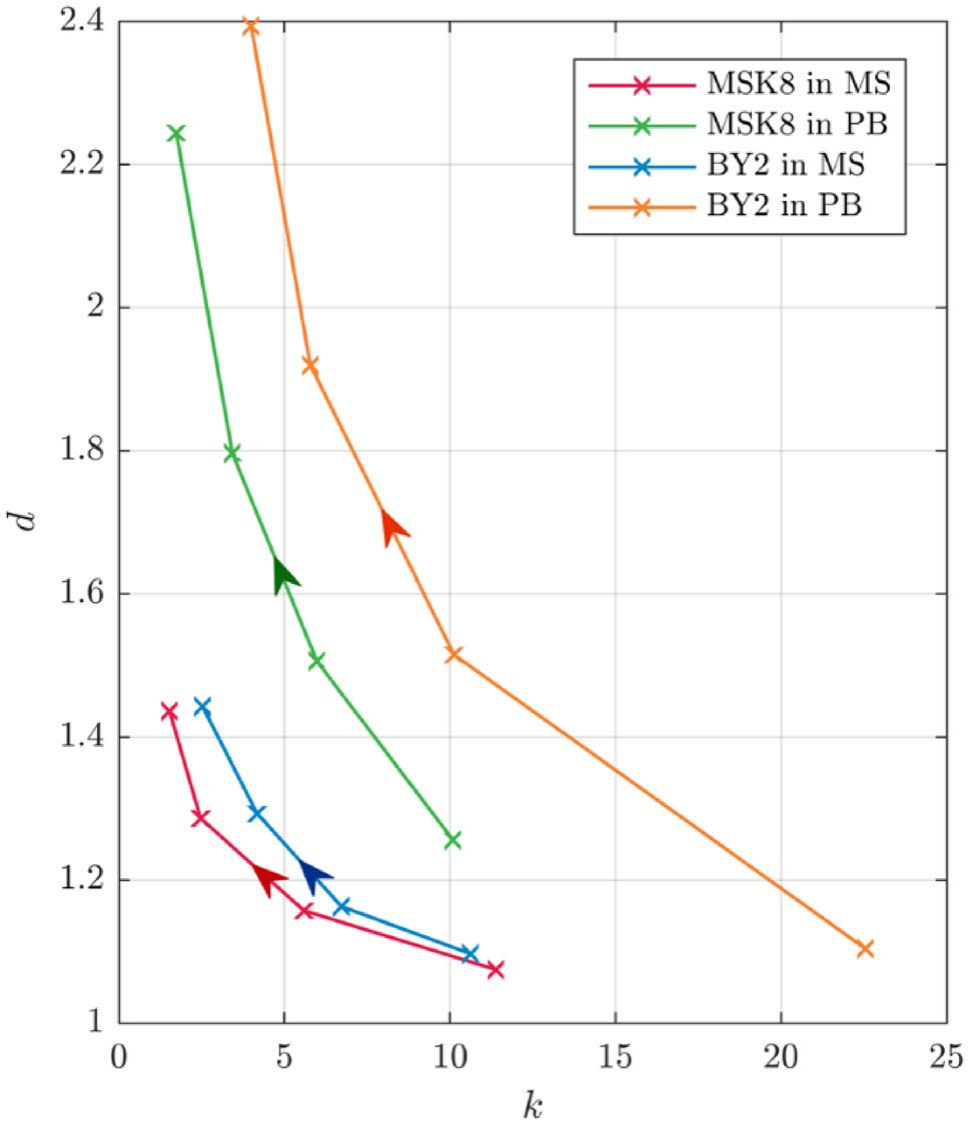
The relationship between $d$ and $k$ for various cell concentrations in both media for both cell types, with the arrows indicating increasing cell concentration.

Hence, in order to amplify the effects of cell concentration on $\left| Z \right|_{5}$, $d$ should be as large as possible. As $d$ is inversely proportional to $\left| Z \right|_{{5{\text{medium}}}}$, the deviation is higher for media with lower conductivity, as shown in Fig. 4. This is consistent with the spectra in Figures S1–S5, where the deviation is larger for PB than for MS.

## Conclusions

In this work, the effect of cell concentrations, cell types and media types of cell suspensions in an ultra-wide frequency range (4 Hz–20 GHz) has been investigated both experimentally and mathematically. The analysis is based on the recently published unified model^33,34^. It was found that as the cell concentration increases, the magnitude of the negative phase peaks ($\varphi_{4} \;{\text{and}}\; \varphi_{6}$) increase. Additionally, as the cell concentration decreases, the minimum phases for a given suspension tend to converge to the intrinsic behavior of the media. Conversely, as the cell concentration increases, the minimum phases converge to the intrinsic behavior of the cells. Furthermore, it was found that $\varphi_{4}$ is determined primarily by the cells (and not the media), and thence the deviation in $\varphi_{4}$ should be lower for suspensions consisting of the same cell type.

It has been mathematically modelled and shown that following the simplified unified impedance model, both $\varphi_{4}$ and $\varphi_{6}$ are independent of the capacitances $C$ and $C_{1}$, and depend only on the ratios of the resistances. For an infinitesimally small $k$, $\varphi_{4}$ approaches − 90° but practically for most cell types and media, $R$ is larger than $R_{1}$. Hence, $\varphi_{4}$ is bounded by approximately − 20° from below. Similarly, as $R_{s}$ is orders of magnitude smaller than $R_{1}$, $\varphi_{6}$ is close to − 90° (and in particular is bounded from above by approximately − 74° for $k = 1$ and $p = 0.01$). Hence, as the scope for improvement is limited by these bounds, in an ideal and best-case scenario, there is room for a 10 × improvement in the sensitivity.

It was further shown that $\varphi_{4}$ depends only on k, whilst the capacitance due to the cells only affects $f_{4}$. It would have been expected that $f_{4}$ would be proportional to the concentration of the cells however, it is found that cell agglomeration has a more significant influence on the capacitance compared to the effect of concentration. On the other hand, $f_{6}$ shows a much clearer relationship on the cell concentration since it depends mostly on the effect of the media (MS or PB). Therefore, in order to amplify the effects of cell concentration on $\varphi_{4}$, $k$ should be as small as possible. Additionally, in order to amplify the effects of cell concentration of $\left| Z \right|_{5}$, d should be as large as possible, hence the deviation is higher for media with lower conductivity.

Hence, this novel approach combines rigorous mathematical analysis with experimental impedance spectral data, laying the foundation for a new kind of sensing methodology. Although at the current stage, the approach only allows for differentiation of cells in suspensions, it has the potential to be extrapolated and employed for monitoring of plant cell expressions in suspensions.

## Supplementary Information

Supplementary Information.
