# Supplementary material for: Towards optimization of plant cell detection in suspensions using impedance-based analyses and the unified equivalent circuit model
Source: Sci Rep. 2021 Sep 29;11:19310. doi: 10.1038/s41598-021-98901-0 (PMC8481493; doi:10.1038/s41598-021-98901-0)
Supplement: Supplementary file 1 — Supplementary Information. [file 41598_2021_98901_MOESM1_ESM.doc]

**Supplementary Materials**

**Towards Optimization of Plant Cell Detection in Suspensions Using Impedance-Based Analyses and the Unified Equivalent Circuit Model**

Kian Kadan-Jamal^a*^^†^, Aakash Jog^c*†^ , Marios Sophocleous^b^, Julius Georgiou^b^, Adi Avni^d^, Yosi Shacham-Diamand^a, c^

^a^ Department of Materials Science and Engineering, Faculty of Engineering, Tel Aviv University, Tel-Aviv 69978, Israel

^b^ Department of Electrical & Computer Engineering, EMPHASIS Research Center, University of Cyprus, Nicosia 1678, Cyprus

^c^ Department of Physical Electronics, School of Electrical Engineering, Faculty of Engineering, Tel Aviv University, Tel-Aviv 69978, Israel

^d^ School of Plant Sciences and Food Security, Tel-Aviv University, Tel-Aviv, Israel

*Corresponding author. Tel.: +972 3 6409549; fax: +972 3 6423508.

^†^These authors contributed equally.

*E-mail address:* kiankadan@mail.tau.ac.il

**Section 1: Comparative Bodes**

Comparative Bodes for single concentration across combination, which were zoomed to see the differences between the cell’s concentrations.

Figure S1-S5, shows the magnitude (top) and phase (bottom) of the Bode plots from 0% to 100% of cell concentration, across all cell-Medium combination.

**
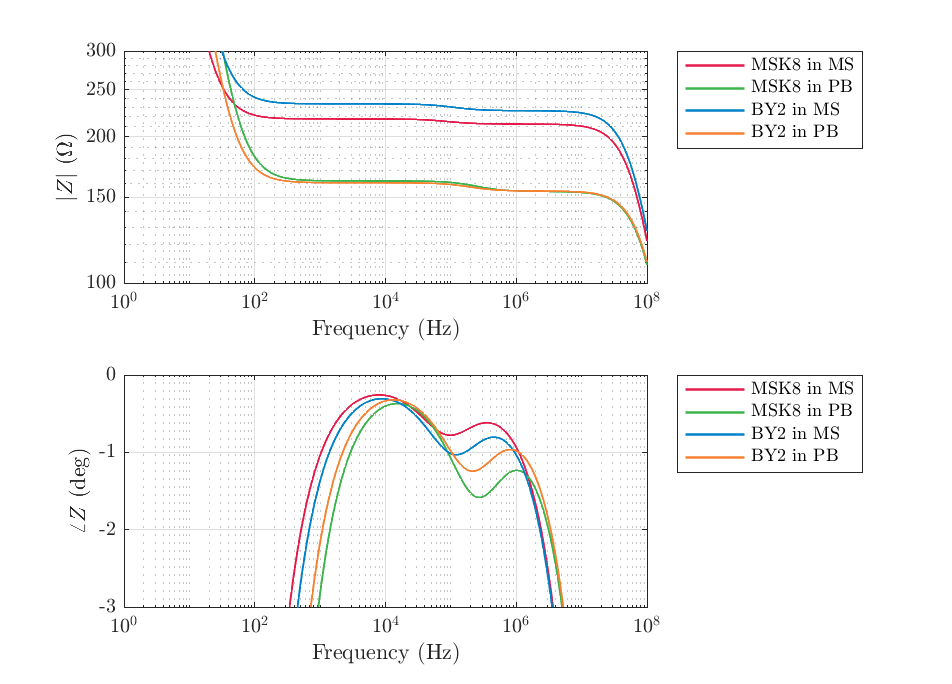
**

**Figure S1.** Bode plots for 0% cell concentration, across all cell-Medium combination.

**
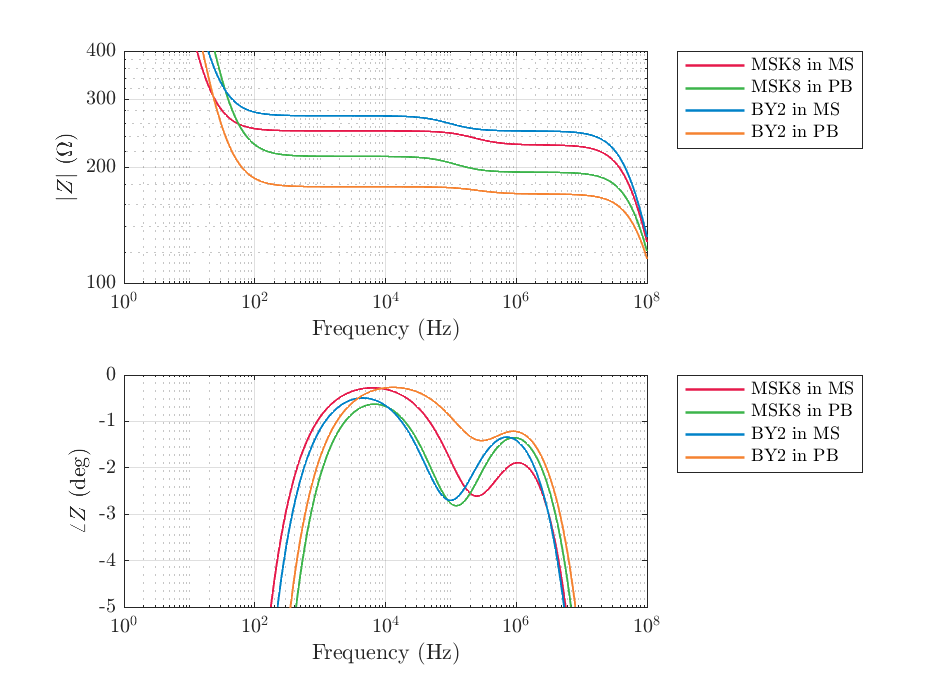
**

**Figure S 2**. Bode plots for 25% cell concentration, across all cell-Medium combination.

**
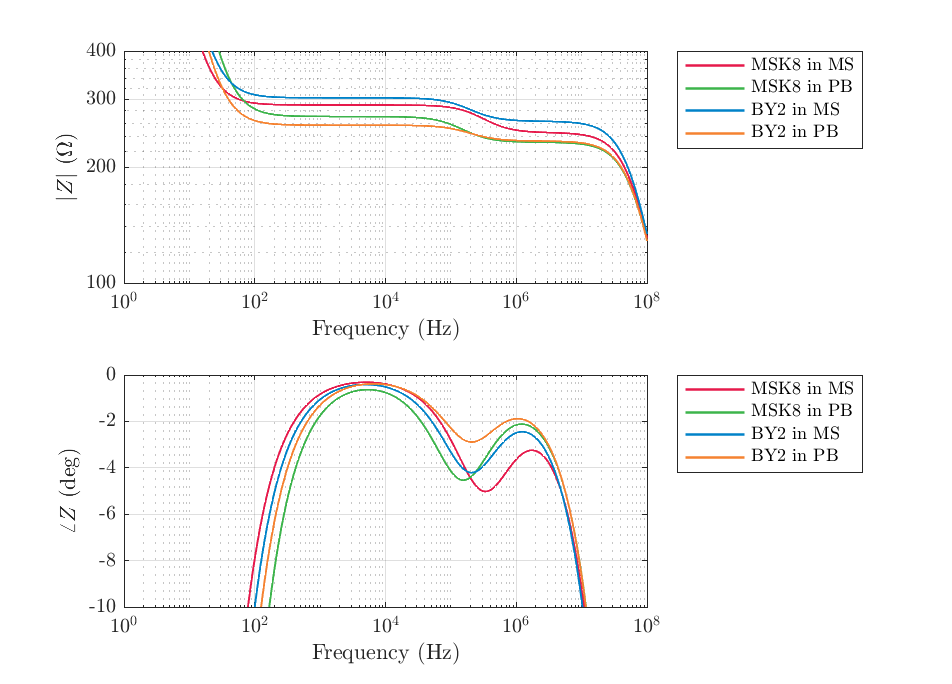
**

**Figure S 3.** Bode plots for 50% cell concentration, across all cell-Medium combination

**
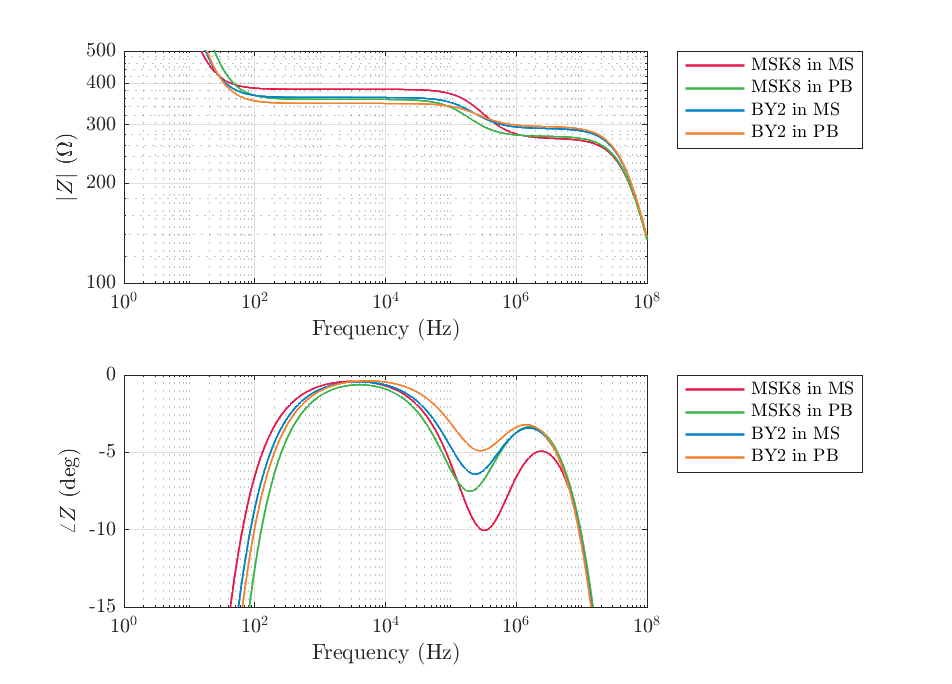
**

**Figure S 4**. Bode plots for 75% cell concentration, across all cell-Medium combination


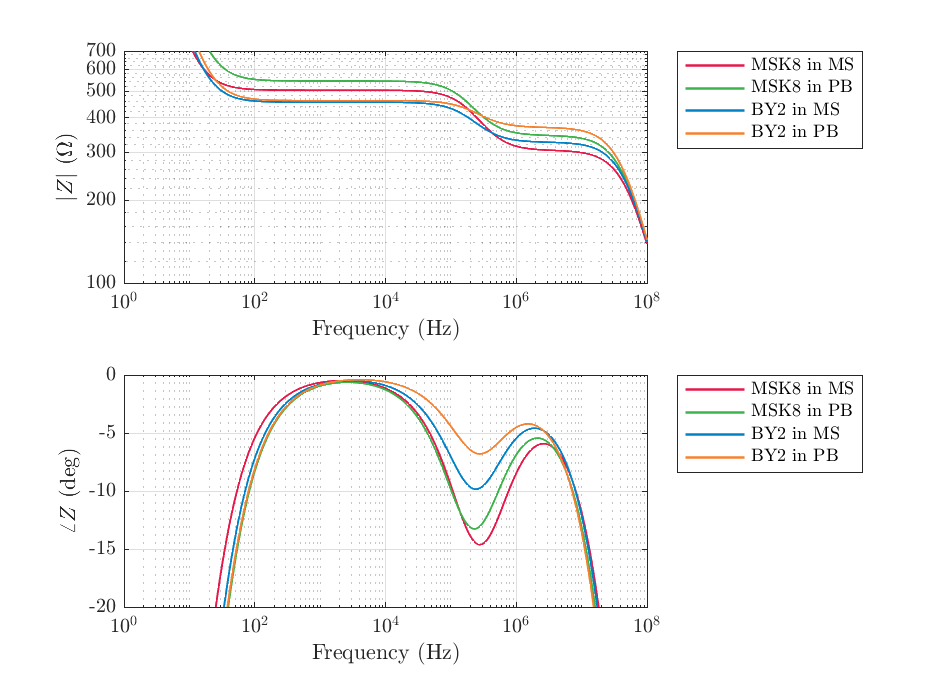


**Figure S 5**. Bode plots for 100% cell concentration, across all cell-Medium combination

**Section 2: Confocal microscopy images analysis**

Confocal microscopy images were acquired using a Zeiss LSM780 confocal microscope system with the Objective LD SC Plan‐Apochromat 20×/1.0 Corr M32.


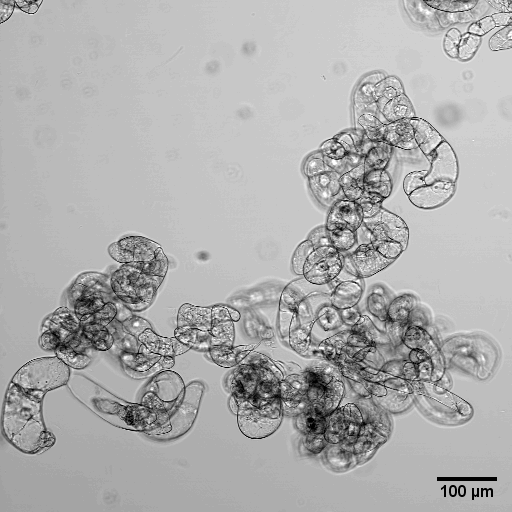

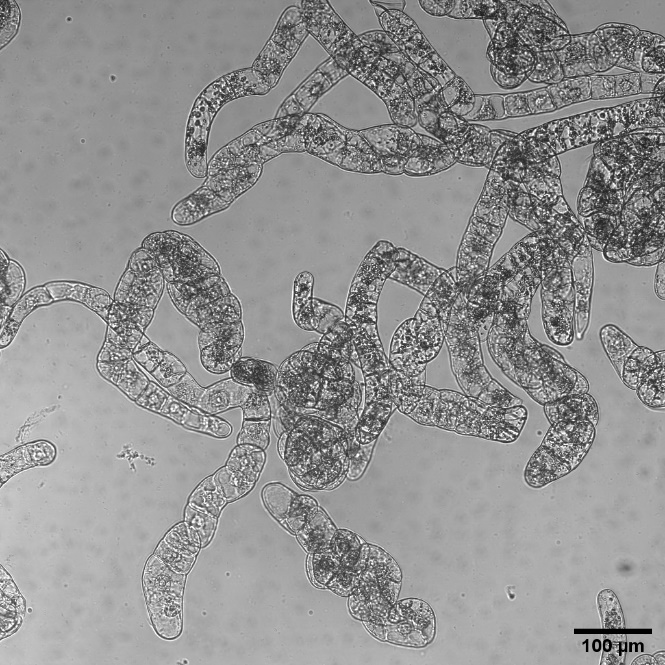


1. (b)

**Figure S 6**. Confocal microscopy images for: (a) tomato- MSK8 cells. (b) tobacco- BY2 cells

**Section 3: Analytical expressions for poles, zeros and impedance magnitudes**

**Table S1:** Analytically obtained poles, zeros and the corresponding impedance magnitudes.

| **Regions (**$\boldsymbol{ij)}$ | $\boldsymbol{\omega}_{\boldsymbol{ij}}$ | $\left\vert\boldsymbol{Z} \right\vert_{\boldsymbol{\omega=}\boldsymbol{\omega}_{\boldsymbol{ij}}}$ |
| --- | --- | --- |
| 1-2 | $\frac{1}{C_{dl}\left( R_{s}+R_{ct}+R_{1} \right)}$ | $\frac{R_{s}+R_{ct}+R_{1}}{\sqrt{2}}$ |
| 2-3 | $\frac{1}{C_{dl}\left( R_{s}+R_{1} \right)}$ | $\left( R_{s}+R_{1} \right)\sqrt{2}$ |
| 3-4 | $\frac{1}{C\left( R_{s}+R_{1} \right)}$ | $\frac{R_{s}+R_{1}}{\sqrt{2}}$ |
| 4-5 | $\frac{1}{CR}$ | $\left( R_{s}+\left( R_{1}\vert\vert R \right) \right)\sqrt{2}$ |
| 5-6 | $\frac{1}{C_{1}\left( R_{s}+(R_{1}\vert\vert R \right)}$ | $\frac{R_{s}+\left( R_{1}\vert\vert R \right)}{\sqrt{2}}$ |
| 6-7 | $\frac{1}{C_{1}R_{s}}$ | $R_{s}\sqrt{2}$ |

**Section 3.1: Derivation of Knee Frequencies between Regions 3 and 4, and Regions 4 and 5**

The total resistance in region 5 is $R_{s}+R_{1}\parallel R$. According to experimental results, $R_{s}$ is two orders of magnitude smaller than $R$ and $R_{1}$. Hence, ignoring $R_{s}$,

$Z=R_{1}\parallel\left( R+Z_{C} \right)=R_{1}\parallel\left( R+\frac{1}{j\omega C} \right){=R}_{1}\parallel\frac{1+j\omega RC}{j\omega C}=\frac{R_{1}\left( 1+j\omega RC \right)}{1+j\omega RC+j\omega R_{1}C}=\frac{R_{1}\left( 1+j\omega RC \right)}{1+j\omega C\left( R+R_{1} \right)}$

Therefore, there exists a zero at $\omega_{45}=\frac{1}{RC}$ and a pole at $\omega_{34}=\frac{1}{\left( R+R_{1} \right)C}$.

**Section 3.2: Derivation of Minimum Phases**

Consider a pole-zero pair where the pole frequency is $\omega_{p}$ and the zero frequency is $\omega_{z}$, such that $\omega_{p}<\omega_{z}$. Therefore, considering the pole in isolation, the phase decreases from $\frac{\omega_{p}}{10}$ to $10\cdot\omega_{p}$, at a rate of -45 deg/dec. Similarly, considering the zero in isolation, the phase increases from $\frac{\omega_{z}}{10}$ to $10\cdot\omega_{z}$, at a rate of +45 deg/dec. Hence, the minimum phase occurs at the midpoint of $\omega_{p}$ and $\omega_{z}$ in logarithmic space, i.e. at $\omega=\sqrt{\omega_{p}\cdot\omega_{z}}$.

Additionally, if the pole is considered in isolation, the phase at a frequency $\omega$ is given by

$\varphi=-arctan \left( \frac{\omega}{\omega_{p}} \right)$ (1)

Similarly, if the zero is considered in isolation, the phase at a frequency $\omega$ is given by

$\varphi=\arctan\left( \frac{\omega}{\omega_{z}} \right)$ (2)

Hence, superposing the phase effects of the pole and the zero,

$\varphi=\arctan\left( \frac{\omega}{\omega_{z}} \right)-\arctan\left( \frac{\omega}{\omega_{p}} \right)$ (3)

Therefore, the minimum phase, i.e. the phase at $\omega=\sqrt{\omega_{p}\cdot\omega_{z}}$ is

$\varphi_{min}=\arctan\left( \sqrt{\frac{\omega_{p}}{\omega_{z}}} \right)-\arctan\left( \sqrt{\frac{\omega_{z}}{\omega_{p}}} \right)$ (4)

**Section 4: Pole frequency dependence on cell concentration**


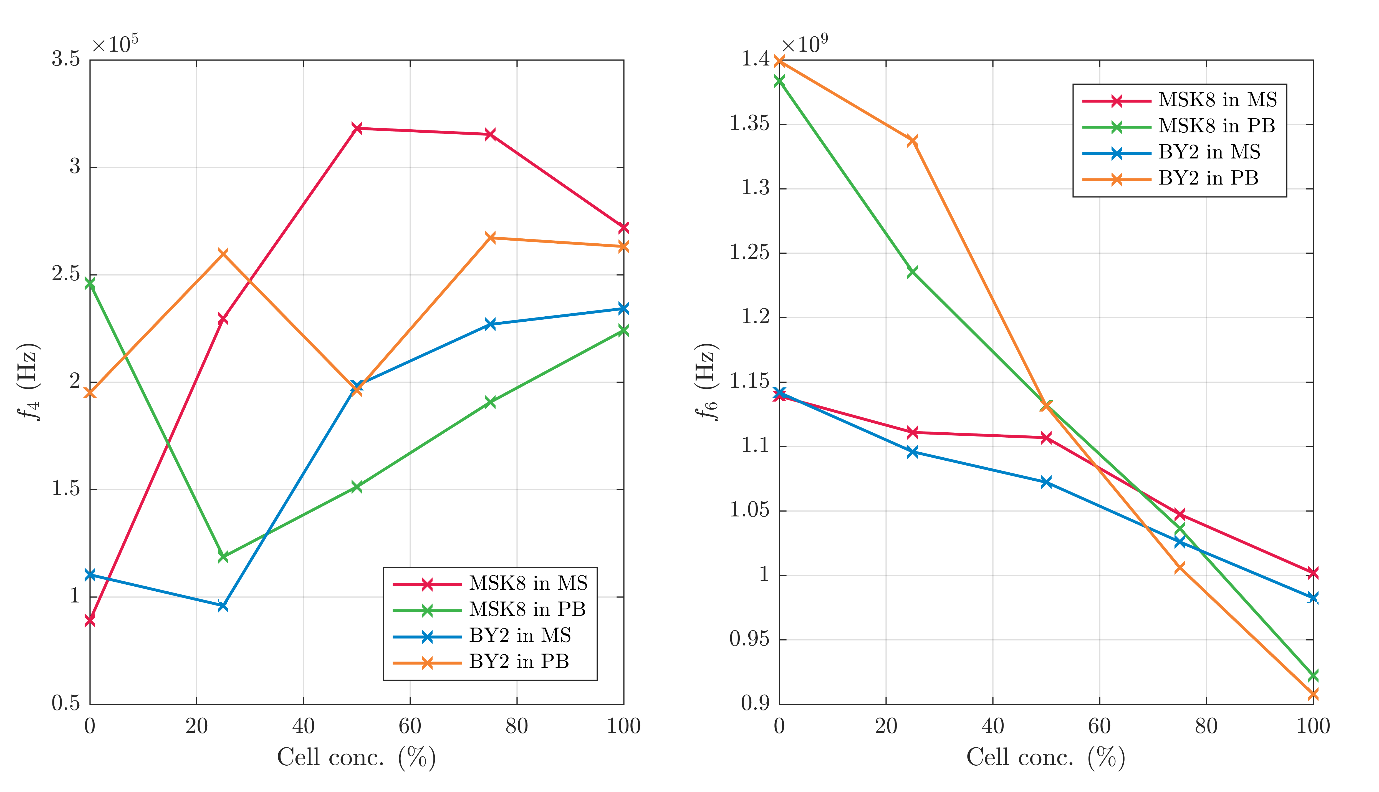
 **Figure S7:** The relationship between pole frequencies and cell concentrations.
